# Supplementary material for: Sonidegib Inhibits the Adhesion of Acute Myeloid Leukemia to the Bone Marrow in Hypoxia: An Optical Tweezer Study
Source: Biomedicines. 2025 Feb 25;13(3):578. doi: 10.3390/biomedicines13030578 (PMC11940413; doi:10.3390/biomedicines13030578)
Supplement: Supplementary file 1 [file biomedicines-13-00578-s001.zip › biomedicines-3346201-supplementary.pdf]

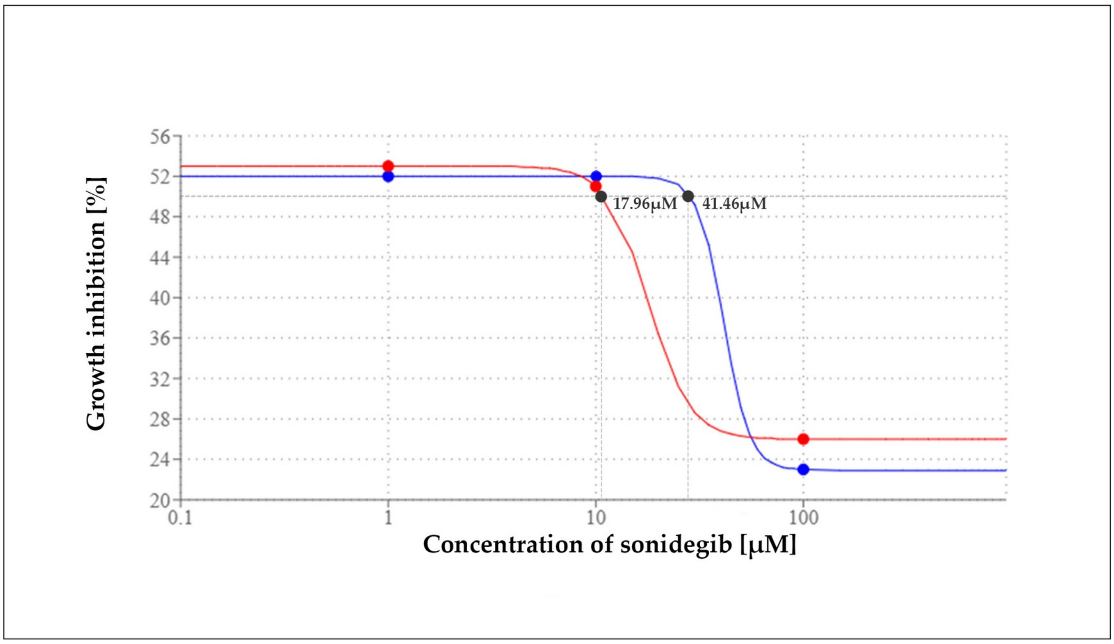

Figure S1. IC50 graphs. The figures show the 50% inhibitory concentrations (IC50) of sonidegib in the OCI-AML3 cell line under two distinct oxygen conditions: atmospheric oxygen (21%) and hypoxic conditions (1%).
